# Conformational Distributions of Phenyl β-D-Glucopyranoside and Gastrodin in Solution by Vibrational Optical Activity and Theoretical Calculations

**DOI:** 10.3390/molecules28104013

**Published:** 2023-05-10

**Authors:** Mutasem Alshalalfeh, Ningjie Sun, Amanda Hanashiro Moraes, Alexandra Paola Aponte Utani, Yunjie Xu

**Affiliations:** 1 Department of Chemistry, University of Alberta, Edmonton, AB T6G 2G2, Canada; alshalal@ualberta.ca (M.A.); b20180539@xs.ustb.edu.cn (N.S.); hanashir@ualberta.ca (A.H.M.); aponteut@ualberta.ca (A.P.A.U.); 2 State Key Laboratory of Advanced Metallurgy, University of Science and Technology Beijing, Beijing 100083, China

**Keywords:** conformational distribution, vibrational circular dichroism, Raman optical activity, phenyl β-D-glucopyranoside, gastrodin, conformational search, DFT calculations

## Abstract

The conformational landscapes of two highly flexible monosaccharide derivatives, namely phenyl β-D-glucopyranoside (ph-β-glu) and 4-(hydroxymethyl)phenyl β-D-glucopyranoside, also commonly known as gastrodin, were explored using a combined experimental and theoretical approach. For the infrared, Raman, and the associated vibrational optical activity (VOA), i.e., vibrational circular dichroism and Raman optical activity, experiments of these two compounds in DMSO and in water were carried out. Extensive and systematic conformational searches were performed using a recently developed conformational searching tool called CREST (conformer-rotamer ensemble sampling tool) in the two solvents. Fourteen and twenty-four low-energy conformers were identified at the DFT level for ph-β-glu and gastrodin, respectively. The spectral simulations of individual conformers were done at the B3LYP-D3BJ/def2-TZVPD level with the polarizable continuum model of the solvents. The VOA spectral features exhibit much higher specificity to conformational differences than their parent infrared and Raman. The excellent agreements achieved between the experimental and simulated VOA spectra allow for the extraction of experimental conformational distributions of these two carbohydrates in solution directly. The experimental percentage abundances based on the hydroxymethyl (at the pyranose ring) conformations G+, G-, and T for ph-β-glu were obtained to be 15%, 75%, and 10% in DMSO and 53%, 40%, and 7% in water, respectively, in comparison to the previously reported gas phase values of 68%, 25%, and 7%, highlighting the important role of solvents in conformational preferences. The corresponding experimental distributions for gastrodin are 56%, 22%, and 22% in DMSO and 70%, 21%, and 9% in water.

## 1. Introduction

Monosaccharides are the basic building units for all carbohydrates and have been shown to provide vital biological functions, from delivering energy for cells to treating a number of genetic disorders [1,2]. Generally, there is a strong connection between the stereochemical properties, including absolute configuration and conformations of a chiral drug molecule and its drug activity. As a result, there has been a great deal of research interest in unravelling the associated chirality and conformational distributions of monosaccharides in solution [3,4].

**Scheme 1:**
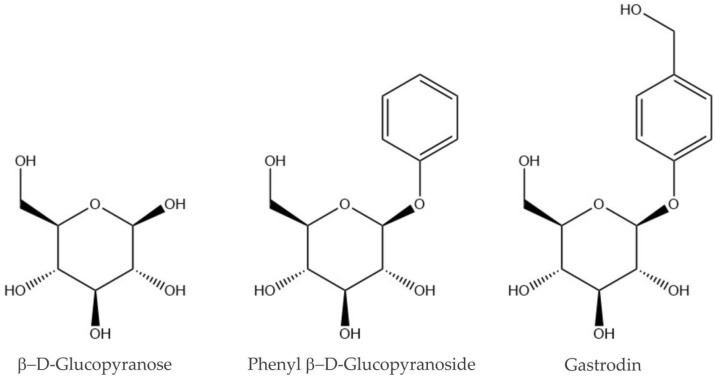
Structures of β-D-glucopyranoside, phenyl β-D-glucopyranoside (ph-β-glu), and gastrodin.

The two monosaccharide derivatives of interest in this work are shown in Scheme 1, namely phenyl β-D-glucopyranoside (ph-β-glu) and 4-(hydroxymethyl)phenyl β-D-glucopyranoside, which is commonly known as gastrodin. Ph-β-glu is a phenolic compound that can be extracted from some medicinally used plants [5]. It has been shown to exhibit anti-inflammatory and anti-cancer activity and has protective effects on the liver when it is exposed to toxic substances [6]. Gastrodin is one main ingredient of *Gastrodia elata* tuber, a traditional Chinese medicine which is used for treating vascular and neurological diseases [7,8]. Although the detailed mechanisms of these bioactivities have not been well understood, some research work in this direction was recently reported. For example, the binding activity of gastrodin to several targets was investigated [9] and the immunomodulatory effects of gastrodin [10] were monitored with a range of tools including near infrared (IR) spectroscopy. These two molecules share the same β-D-glucopyranoside subunit, which is also depicted in Scheme 1. While ph-β-glu has a benzene substituent at the anomeric position, gastrodin has a phenyl-para-methanol substituent at the anomeric position.

The possible conformations of ph-β-glu in the gas phase were first investigated by Talbot and Simons [11] by using mass-selected resonant two-photon ionization and IR ion dip spectroscopy where the authors identified three low-energy conformers of the β-anomer which all show the exocyclic hydroxyl group in the equatorial position. Later, Simons and co-workers reported that the addition of just one water molecule was enough to drastically change the conformational landscape of ph-β-glu and discussed the implications of this for measurements done in solution [12]. In searching for vibrational circular dichroism (VCD) markers for differentiation of the α- and β-anomeric configuration in solution, Monde and co-workers [13] surveyed the experimental IR and VCD spectra of several naturally occurring monosaccharides, including ph-β-glu and ph-α-glu in deuterated-dimethyl sulfoxide (DMSO) solution. They identified an intense, sharp, and negative VCD signature at about 1145 cm^−1^ as a marker for the α-D-anomers, whereas no such strong feature was observed for the β-D-anomers. For gastrodin, no similar conformational or chiroptical spectroscopic investigations have been reported in the gas phase or in solution so far.

The vibrational optical activity (VOA) spectroscopies have emerged in the last ten years as powerful spectroscopic tools for determining absolute configuration and dominating conformations of a wide range of chiral molecules directly in solution, such as amino acids [14,15], peptides [16], proteins [17], and carbohydrates [18,19]. These methods include VCD [20], Raman optical activity (ROA) [21], and a recently discovered new form of chiral Raman spectroscopy [22,23], which has already been utilized in several studies of chiral molecules under (near) resonance [24]. To extract stereochemical properties, including chirality and conformational distributions from the VOA spectroscopic data, it is essential to utilize an efficient strategy to identify all possible low-energy conformers of the molecular targets. The recently developed conformer-rotamer ensemble sampling tool (CREST) by Grimme and co-workers [25] has emerged as an effective and efficient conformational search tool. CREST has been successfully utilized and at the same time benchmarked in rotational spectroscopic studies of flexible molecules [26] and non-covalently bonded clusters [27,28] where rotational spectra of individual conformers can be identified separately, in contrast to most condensed phase studies. So far, the only documented failure of CREST in producing a conformational candidate is the case of the heterochiral trimer of propylene oxide [29] whose geometry was established experimentally by rotational spectroscopic data of different isotopologues. More recently, CREST has been utilized in several VOA studies, for example, the recent report of conformations of steroid hormones by VCD [30] and the development of a computational protocol for VCD spectra of cyclic oligopeptides [31].

Since no conformational distribution investigations have been reported for ph-β-glu and gastrodin in solution directly, we applied a combined experimental and theoretical approach by using both VCD and ROA spectroscopies to extract conformational distributions of these two chiral molecular targets in DMSO and in water. First, we aimed to systematically identify all possible low-energy conformers of ph-β-glu and gastrodin by applying CREST [24]. The subsequent DFT calculations were carried out to facilitate theoretical IR, VCD, Raman, and ROA simulations and comparisons with the experimental data. Second, the influence of conformations on the resulting VCD and ROA spectral features was discussed in detail, since these VOA features are much more sensitive to small structural changes than their parent spectroscopies. Finally, the conformational distributions of ph-β-glu and gastrodin in DMSO and in water were obtained and compared and were also compared to those obtained in the gas phase whenever available to appreciate the effects of solvents on conformational preferences.

## 2. Results and Discussion

In the following, we first describe the general conformational searches and DFT geometry optimizations performed for ph-β-glu and gastrodin and summarize all the low-energy minima identified in DMSO and in water in Section 2.1. Next, comparisons of the simulated and experimental IR and VCD spectra of ph-β-glu and of gastrodin in DMSO are discussed in Section 2.2. In Section 2.3, the experimental and theoretical Raman and ROA spectra of ph-β-glu and of gastrodin in water are explained. Overall, the good agreements between experiment and theory allow one to conclusively identify the main conformers of ph-β-glu and gastrodin in these two solvents. Finally, we examined how the conformational preferences alter from the gas phase to DMSO and to water and compared their conformational distributions in Section 2.4.

### 2.1. Low-Energy Conformers of Phenyl β-D-Glucopyranoside and Gastrodin

**Figure 1:**
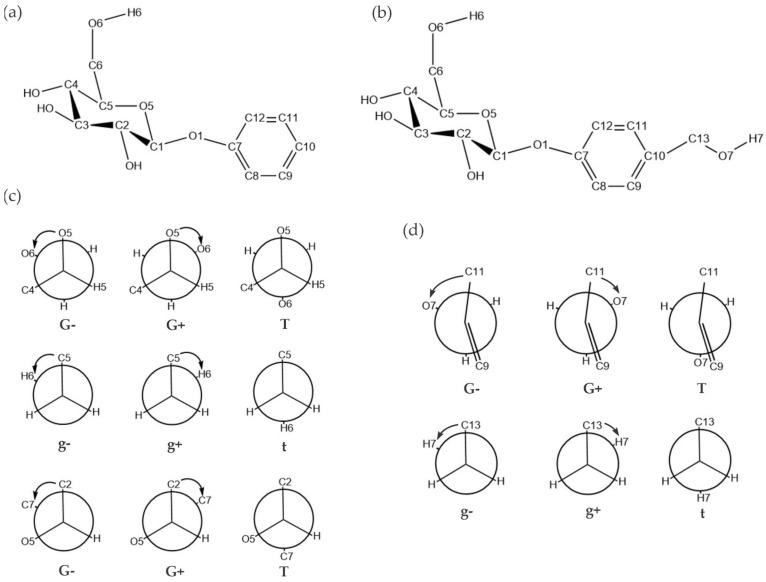
The structural formula of (**a**) ph-β-glu and (**b**) gastrodin where both molecules are shown in the ^4^C_1_ chair conformations. (**c**) Newman projections of the plausible conformations of the hydroxymethyl group of the pyranose ring around the C5–C6 and C6–O6 bonds and the plausible conformations of the phenyl group around the C1–O1 bond. (**d**) Newman projections of the plausible conformations of the hydroxymethyl group of the phenyl ring around the C10–C13 and C13–O7 bonds.

**Figure 2:**
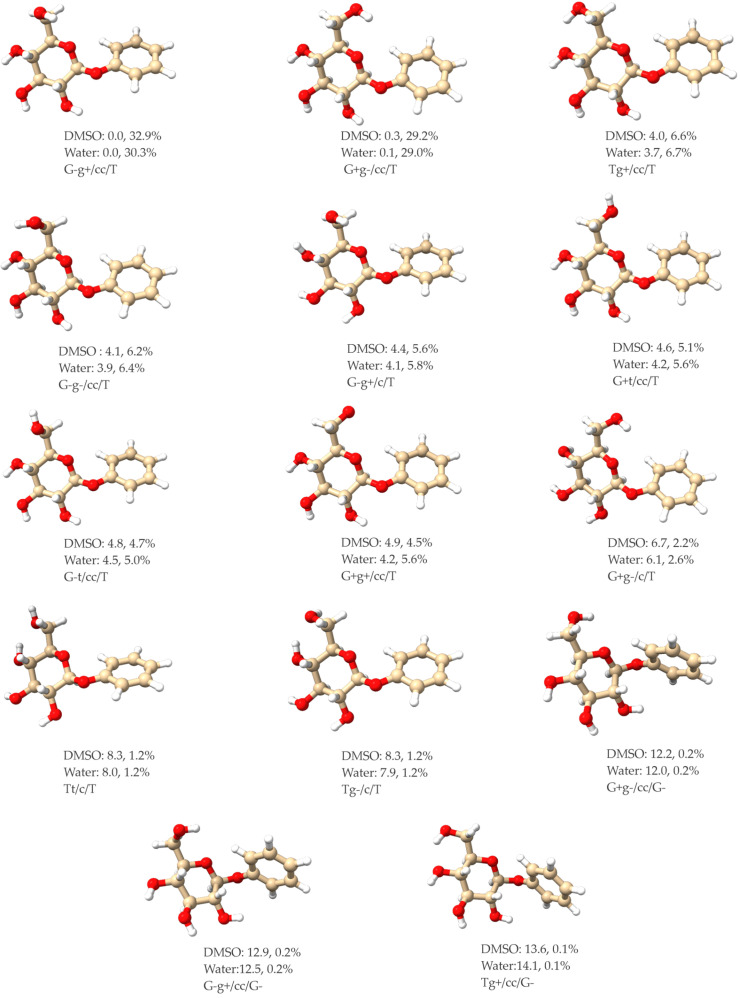
Geometries of the 14 most stable conformers of ph-β-glu at the B3LYP-D3BJ/def2-TZVPD level with the PCM of DMSO and water. The relative free energies in kJ mol^−1^ and the percentage Boltzmann factors at 298 K are also given.

As can be seen in Scheme 1, β-D-glucose, ph-β-glu, and gastrodin share the same pyranose ring. To facilitate the discussion of the conformational geometries, we first introduced the naming scheme, similar to those used in the rotational spectroscopic investigation of β-D-glucose [32] and in the previous literature [12]. Briefly, the letter β was used to describe the anomer type where the exocyclic O group at the anomeric carbon (C1) and the hydroxymethyl group are on the same face of the ring. Since one does not expect a conversion between the β and the α anomers for the substituted glucoses such as ph-β-glu and gastrodin, we do not include this part in the conformational naming for conciseness. For ph-β-glu, the naming starts with the conformation of the pyranose ring hydroxymethyl group which is labelled with G+, G-, or T for the torsion angle O6–C6–C5–O5 of about 60°, −60°, or 180°, respectively, and g+, g-, or t for the torsion angle H6–O6–C6–C5 in the same way (See Figure 1). Next, the ring hydroxyl groups in the lower-energy conformers typically form a counter-clockwise (cc) or clockwise (c) chain of (weak) cooperative network of intramolecular hydrogen bonding type contacts, as reported in the previous studies [12,31]. Furthermore, the phenyl group attached to O1 may rotate about the C1–O1 bond to generate several torsional conformations. Again, we label them as G+, G-, or T where the torsion angle C2–C1–O1–C7 is about 60°, −60°, or 180°, respectively. Finally, the phenyl plane may tilt differently by rotating about the O1–C7 bond. The systematic names are given in Figure 2 to identify the specific conformers of ph-β-glu. For example, the global minimum in DMSO (vide infra) is G-g+/cc/T where the ring hydroxymethyl group, G-g+, is listed first, followed by a slash and the symbol for the OH···O contact directions of the ring hydroxyl groups, i.e., cc, and finally after a second slash, the phenyl torsional conformation T. Regarding the tilting of the phenyl plane, the corresponding C1–O1–C7–C12 dihedral angle varies from about 13° to 16° for conformers with the T phenyl torsional conformation, and from −51° to −52° for the G- phenyl torsional conformation, respectively (See Figure S1, Supplementary Materials for the detailed dihedral angle values). For conciseness, we decided not to further label the conformation associated with the tilting of the phenyl plane because it has a one-to-one relationship with the phenyl torsional conformation.

**Figure 3:**
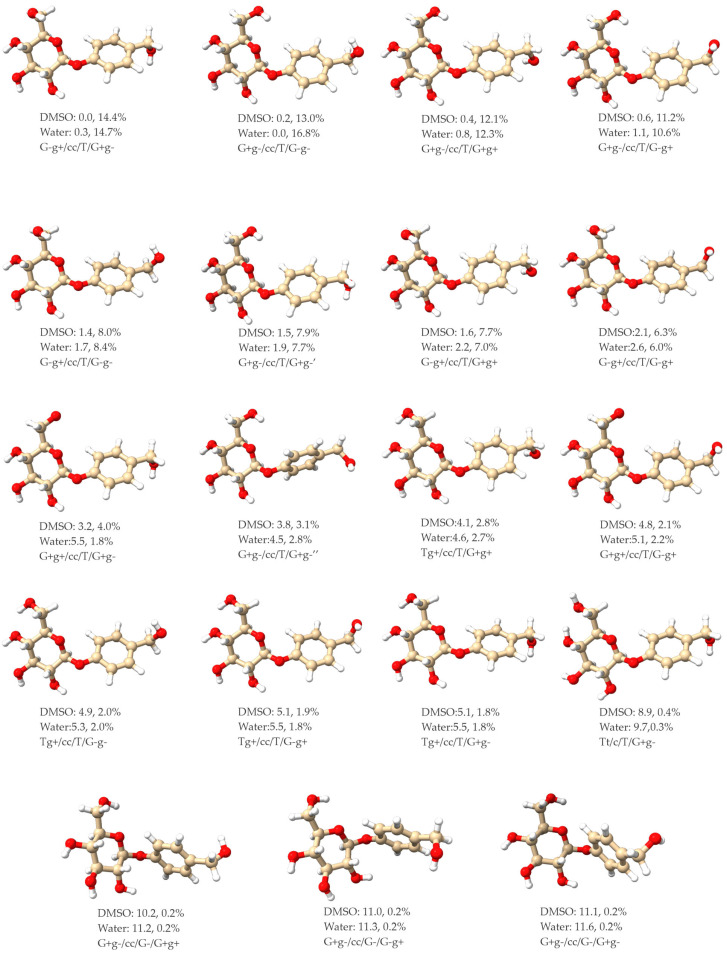
Geometries of the 19 most stable conformers (i.e., conformers with Boltzmann population > 0.1%) of gastrodin at the B3LYP-D3BJ/def2-TZVPD level with the PCM of DMSO and Water. The relative free energies in kJ mol^−1^ and the percentage Boltzmann factors at 298 K are also listed. Note that conformer No. 6 and 10 have the same name based on the current scheme but have different phenyl group titling positions and are differentiated by adding ‘and’ at the end.

For gastrodin, an additional label is needed for the hydroxymethyl group attached in the para position to the phenyl group is needed. Here, we use G+, G-, or T when the torsion angle C11–C10–C13–O7 is about 60°, −60°, or 180°, respectively, and g+, g-, or t are similarly defined for the torsion angle C10–C13–O7–H7 for the phenyl hydroxymethyl group. For example, the global minimum of gastrodin in DMSO (vide infra) is G-g+/cc/T/G+g- where the first three units describe the conformation of the ph-β-glu part. As described above, a further slash followed by G+g- describes the conformation of the hydroxymethyl group at the phenyl ring (see Figure 1d). These systematic names are used in Figure 2 and Figure 3 to identify the specific conformers of ph-β-glu and gastrodin. The C1–O1–C7–C12 dihedral angle values which correspond to the tilting degrees of the phenyl plane are given in Figure S2, Supplementary Materials.

We applied CREST for systematic conformational searches of ph-β-glu and gastrodin with the GFN2-xTB GBSA [33] implicit solvation model, where GBSA stands for the generalized Born (GB) model with surface area (SA) contributions [34]. An energy cut-off window of 60 kJ mol^−1^ was applied for the CREST searches, leading to several hundred structural candidates for ph-β-glu and gastrodin in DMSO and water. We further utilized a previously developed multitier approach [35] to potentially capture all low-energy conformers within an energy window of 15 kJ mol^−1^ before the final DFT geometry optimizations and spectral simulations at the B3LYP [36,37]-D3BJ/def2-TZVPD [38] level. Here, the D3 dispersion correction [39,40] with the Becke–Johnson (BJ) damping function [41] was utilized. The details of the CREST and the DFT calculations are described in Section 3. Materials and Methods.

Initially, we applied the CREST searches in the gas phase and then carried out the final geometry optimizations in the respective solvents, i.e., DMSO and H_2_O. This approach caused some issues in the DFT geometry optimization step because some CREST geometries were too far from the final optimized geometries and extra time was needed to reach convergence. We therefore redid the CREST searches in the respective solvents directly. The above procedures resulted in fourteen low-energy conformers of ph-β-glu. For gastrodin, which has an additional -CH_2_OH substitution, twenty-four low-energy conformers were identified. Overall, similar sets of conformers of ph-β-glu were identified in DMSO and in water, although their relative energy ordering was altered in some cases. A similar conclusion can be drawn for the conformers of gastrodin in DMSO and in water. The resulting geometries, relative free energies, and percentage Boltzmann factors at room temperature of the ph-β-glu conformers are summarized in Figure 2. The corresponding results of the 19 gastrodin conformers with a percentage Boltzmann factor > 0.1% are given in Figure 3, while the five less stable gastrodin conformers whose Boltzmann factors are ≤0.1% are depicted in Figure S3, Supplementary Materials).

Some general observations can be made about these low-energy structures of both molecules in DMSO and in water. First, all their pyranose rings take on the ^4^C_1_ chair configuration. This is expected since the boat configuration tends to be in the order of 40 kJ mol^−1^ less stable than the chair global minimum [12]. Second, the three equatorial hydroxyl groups at C2, C3, and C4 of the glucose ring form a chain of weak intramolecular attractive OH···O contacts, linking each hydroxyl group to its neighbor with a typical OH···O distance of about 2.5 Å (see Table 1 and Table 2). In all the conformers present in Figure 2 and Figure 3, these contact networks either take on the cc or c arrangements. Furthermore, in the most preferred conformations of both ph-β-glu and gastrodin, the ring hydroxyl groups take on the cc network, whereas those with the c hydrogen contact networks appear in the (much) less preferred conformations.

For the ph-β-glu conformers in DMSO, one can roughly classify them into four groups based on their relative energies. (i) This group includes the global minimum (G-g+/cc/T) and the next nearly iso-energy (i.e., ΔG = 0.3 kJ mol^−1^) conformer (G+g-/cc/T); (ii) The second group is about 4~5 kJ mol^−1^ less stable and mainly includes a set of cc conformers, Tg+/cc/T, G-g-/cc/T, G+t/cc/T, G-t/cc/T, and G+g+/cc/T, as well as one c conformer G-g+/c/T; (iii) The next group is about 7~8 kJ mol^−1^ higher in energy than those in (i) and consists of only c-conformers, G+g-/c/T, Tt/c/T, and Tg-/c/T; and (iv) The final groups were about 12~14 kJ mol^−1^ with G+g-/cc/G-, G-g+/cc/G-, and Tg+/cc/G-. A similar classification can be made for the ph-β-glu conformers in water, even though the relative energy ordering of the ph-β-glu conformers in water within a group are often altered somewhat from those in DMSO. Some important structural parameters of the four most stable ph-β-glu conformers in DMSO are summarized in Table 1, while the corresponding ones in water are listed in Table S1, Supplementary Materials. The structural values in DMSO and in water are very similar where the largest change in bond lengths is ~0.007 Å and the largest change in angles is 0.3°.

In comparison to ph-β-glu, the additional -CH_2_OH substituent in the para position of the phenyl group in gastrodin increases the number of low-energy conformers significantly and blurs the energy separations for the four ph-β-glu groups discussed above. For example, while the most stable gastrodin conformers still adopt the G-g+/cc/T or G+g-/cc/T ph-β-glu cores, their free energy difference can be as large as 3.6 kJ mol^−1^ between G+g-/cc/T/G-g- and G+g-/cc/T/G+g-‘’, highlighting the importance of the phenyl hydroxymethyl conformation on the overall stability. Some important structural parameters of the four most stable gastrodin conformers in DMSO are listed in Table 2 to facilitate the differentiation of these conformers, while the corresponding values in water are given in Table S2, Supplementary Materials. The structural values of gastrodin in DMSO and in water exhibit more noticeable differences than ph-β-glu. For example, the largest change in bond lengths is ~0.02 Å and in angles it is 2.0° for G-g+/cc/T/G+g-.

### 2.2. Experimental and Simulated IR and VCD Spectra of ph-β-Glu and Gastrodin

**Figure 4:**
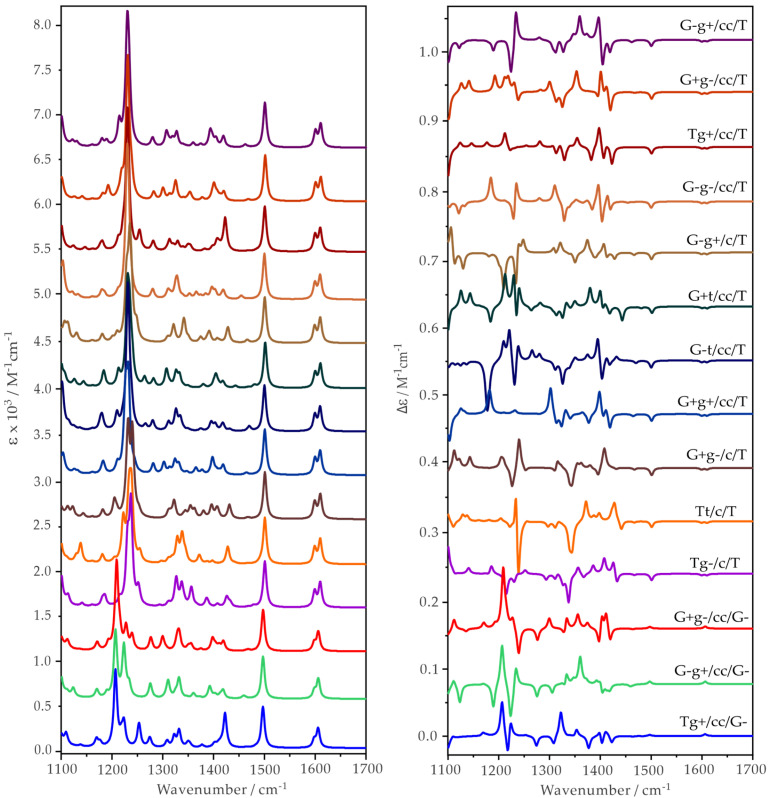
The simulated individual IR and VCD spectra of the ph-β-glu conformers at the B3LYP D3BJ/def2-TZVPD level with the PCM of DMSO. The conformers are shown based on their theoretical stability, with the most stable one at the top to the least stable one at the bottom.

The individual IR and VCD spectra of the most stable ph-β-glu conformers shown in Figure 4 were simulated at the B3LYP-D3BJ/def2-TZVPD level. The simulated IR and VCD spectra of the conformers are essentially identical, indicating that the addition of the extra dispersion function provided little difference. We therefore present only the results obtained at the B3LYP-D3BJ/def2-TZVPD level. The individual conformer IR and VCD spectra of ph-β-glu are depicted in Figure 4. It is interesting to note that while the simulated IR spectra of the individual conformers appear similar, their VCD features differ greatly. For example, the VCD features in the vicinity of 1200 cm^−1^, which mainly correspond to the wagging motions of the CH and OH groups of the pyranose ring, vary drastically from one conformer to the next. This significant variation highlights the high sensitivity of VCD features to small conformational changes, providing an effective tool to extract experimental conformational distributions in solution directly.

**Figure 5:**
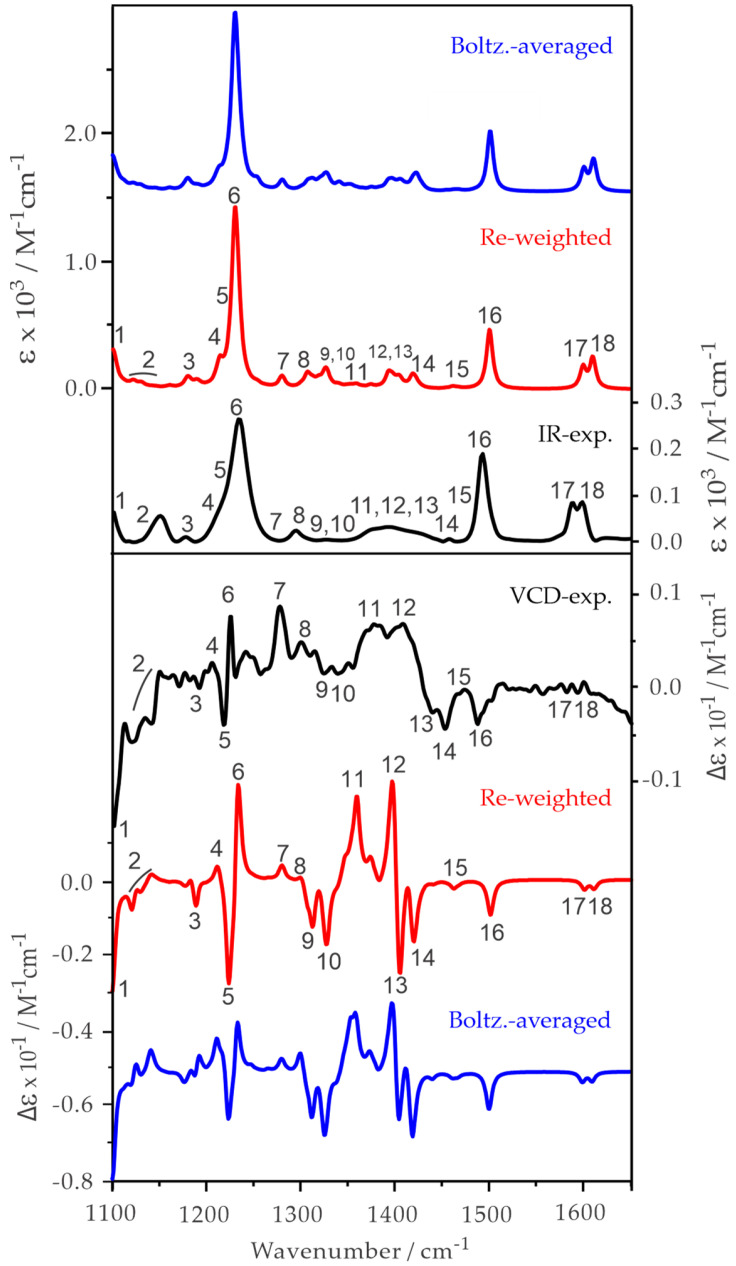
Comparison of the experimental IR and VCD spectra of ph-β-glu in DMSO with the corresponding Boltzmann averaged and re-weighted IR and VCD spectra, simulated at the B3LYP-D3BJ/def2-TZVPD level with the PCM(DMSO). The predicted Boltzmann percentage factors are listed in Figure 2 and the adjusted Boltzmann factors are described in the main text. The main IR and VCD features are numbered to facilitate easy comparison.

To analyze the experimental spectra, we first compared the Boltzmann averaged IR and VCD spectra of the 14 most stable conformers with the experimental spectra in Figure 5, using the Boltzmann population factors provided in Figure 2. In general, the Boltzmann average IR and VCD spectra exhibit an acceptable agreement with the experimental data. On the other hand, the main experimental features in the 1200–1300 cm^−1^ region are not as well captured, especially the bisignate VCD, which was centered at about 1229 cm^−1^. It is well accepted in the IR and VCD community that the vibrational spectral features predicted are generally quite robust, whereas the relative free energy values are more difficult to reliably predict. A close examination of the individual conformer spectra and the experimental ones in Figure 3 indicates that the global minimum, G-g+/cc/T, exhibits the VCD pattern closely resembling the experimental one, whereas the simulated VCD pattern of the second main conformer, G+g-/cc/T, deviates greatly from the experimental one in the 1200–1300 cm^−1^ region. Taking into account both the IR and VCD comparison and the general stability trend predicted, we arrived at the empirical weights of 60% for G-g+/cc/T, 10% for G+g-/cc/T, Tg+/cc/T, and G-g-/cc/T, and 2.5% for the next four most stable conformers: G-g+/c/T, G+t/cc/T, G-t/cc/T, and G+g+/cc/T. The resulting re-weighted IR and VCD based on the empirical weights obtained above are also presented in Figure 5.

The re-weighted IR and VCD spectra show a very good agreement with the experimental data. Below, we discuss some specific features in more detail. By increasing the Boltzmann percentage factor of the global minimum, G-g+/cc/T, and reducing that of the second conformer, G+g-/cc/T, the experimental negative/position bisignate features, labelled as 5 and 6, are now much better captured. In fact, the VCD features below 1300 cm^−1^ are better reproduced overall by the re-weighted spectrum. For example, VCD band 7 is now more intense than VCD band 8, consistent with the experimental observation. It is interesting to note that the third most stable conformer, Tg+/cc/T, contributes noticeably to this feature 7, and its weight was raised in the re-weighting scheme.

**Figure 6:**
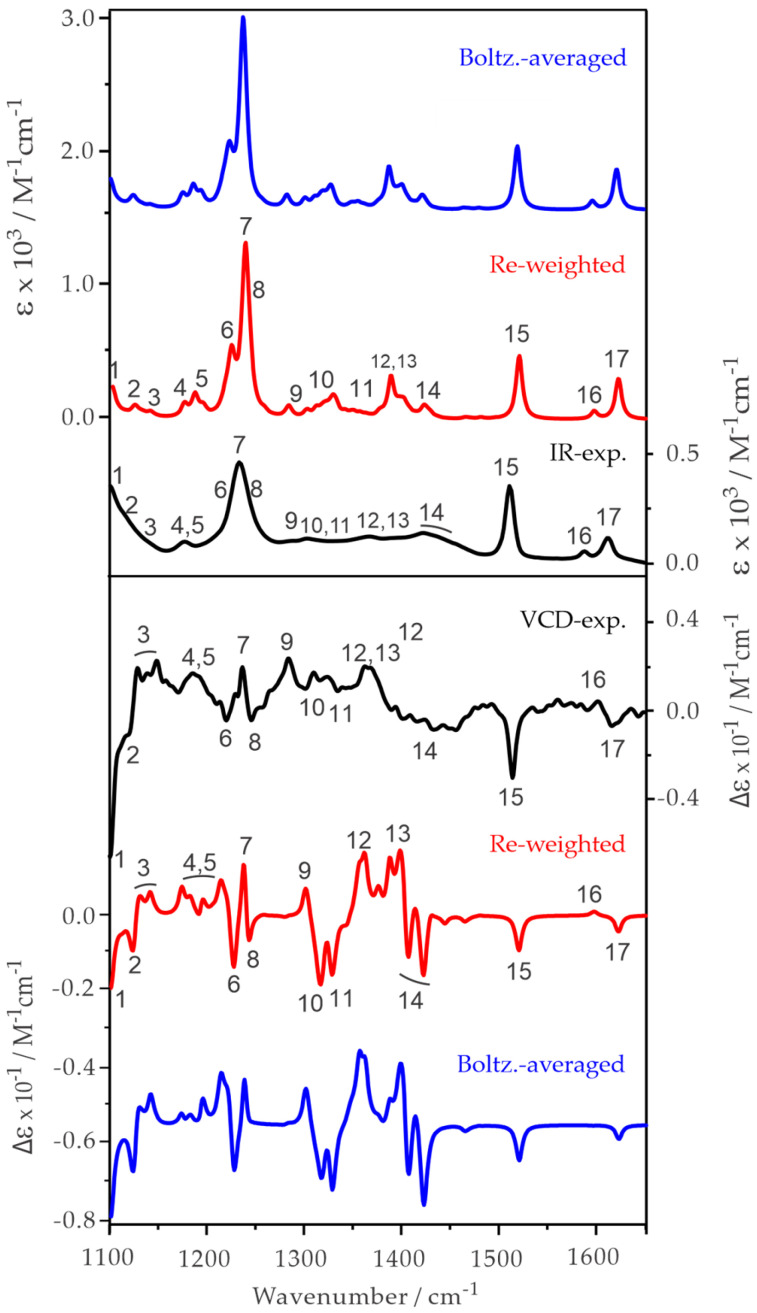
Comparison of the experimental IR and VCD spectra of gastrodin with the corresponding Boltzmann averaged IR and VCD spectra at the B3LYP-D3BJ/def2-TZVPD level with the PCM of DMSO theory at 298 K, and the re-weighted IR and VCD spectra. The percentage Boltzmann factors are given in Figure 3, while the empirical ones are provided in the main text. The main IR and VCD features are numbered to facilitate easy comparison.

For the analysis of the IR and VCD spectra of gastrodin in DMSO, we applied a very similar approach to that described above. The individual IR and VCD spectra of the 19 most stable gastrodin conformers are shown in Figure S4, Supplementary Materials, while those of the five least stable conformers with percentage abundances ≤ 0.1% are not shown. The IR spectra of individual gastrodin conformers are generally quite similar, whereas the corresponding VCD spectra differ greatly, offering an opportunity to extract the conformational distribution of gastrodin in solution. To analyze the experimental spectra, we first compared the Boltzmann averaged IR and VCD spectra of the nineteen most stable conformers with the experimental spectra in Figure 6.

In general, the Boltzmann average IR and VCD spectra exhibit an acceptable agreement with the experimental data of gastrodin. When one examines the individual VCD spectra of the gastrodin conformers, the VCD spectral features of Tt/c/T/G+g- seem to largely resemble the observed VCD features in the 1200–1300 cm^−1^ region. We therefore re-adjusted the percentage abundances to reflect the contribution of Tt/c/T/G+g-. Since there are a large number of possible conformers, we simplified the process by using the same percentages for those with similar stabilities and left out those with percentages less than 0.5%. Taking into account the IR, VCD comparison, and the general conformational stability trend, we arrived at the empirical weights of 14% for G-g+/cc/T/G+g- and 6% for the next three conformers: G+g-/cc/T/G-g-, G+g-/cc/T/G+g+, and G+g-/cc/T/G-g+, 8% for the next six conformers: G-g+/cc/T/G-g-, G+g-/cc/T/G+g-‘, G-g+/cc/T/G+g+, G-g+/cc/T/G-g+, G+g+/cc/T/G+g-, and G+g-/cc/T/G+g-‘’, 2% for the next five conformers: Tg+/cc/T/G+g+, G+g+/cc/T/G-g+, Tg+/cc/T/G-g-, Tg+/cc/T/G-g+, and Tg+/cc/T/G+g-, and finally 15% Tt/c/T/G+g-. The resulting re-weighted IR and VCD are also presented in Figure 6. The main observed VCD features and the corresponding simulated ones were numbered to facilitate easier comparison. As one can see, the re-weighted VCD spectrum improves the agreement with the experiment in the 1200–1300 cm^−1^ noticeably, while the agreements in other regions remain of similar quality to the Boltzmann weighted one.

**Figure 7:**
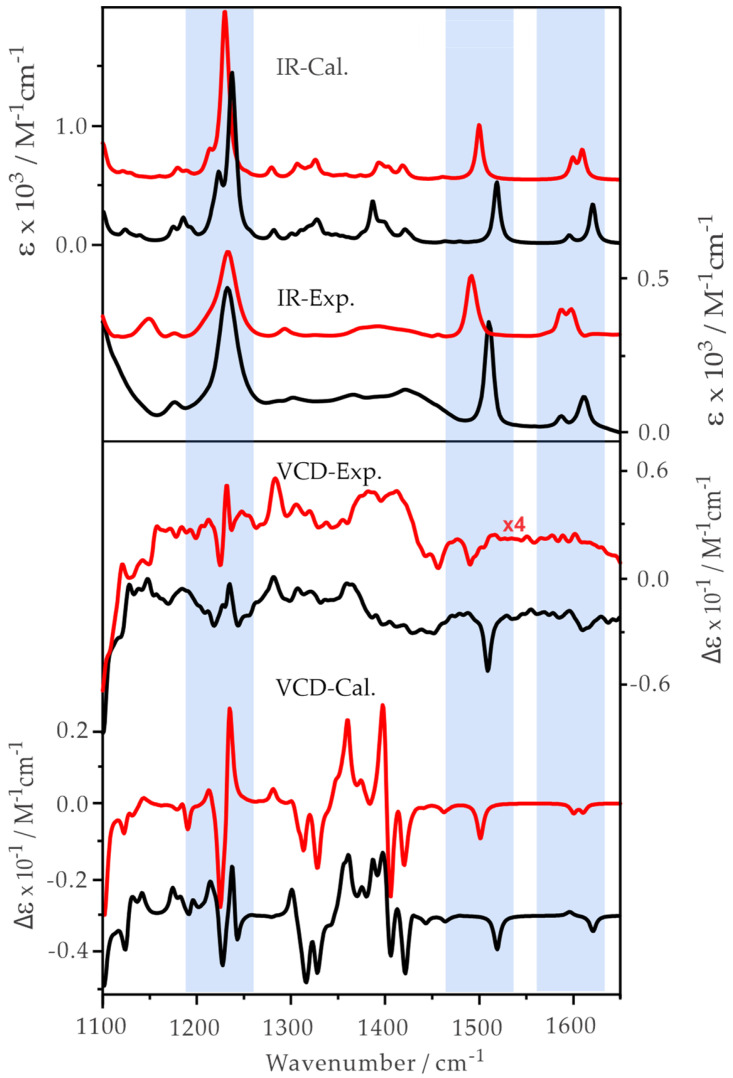
Comparison of the experimental IR and VCD spectra of ph-β-glu and gastrodin with the corresponding re-weighted simulated IR and VCD spectra at the B3LYP-D3BJ/def2-TZVPD level with the PCM of DMSO. Several regions are shaded to draw attention to the changes in the IR and VCD features in the experiments and how well they were reproduced by the modelling.

Since ph-β-glu and gastrodin differ only in the extra hydroxymethyl group at the para position at the phenyl ring for the latter, one may expect some similarity and/or relationships of their IR and VCD bands. How well are these systematic changes captured by the current theoretical modelling? In Figure 7, we provide a comparison of the experimental and theoretical IR and VCD of these two compounds. Three regions are highlighted in the figure, which are centered at about 1220, 1500, and 1600 cm^−1^. The IR and VCD pattern changes from ph-β-glu to gastrodin in the 1600 cm^−1^ region, corresponding to the C=C stretching modes of the phenyl ring, are well reproduced theoretically. Similar statements can be made for the other two shaded regions, supporting the interpretation of the experimental results.

### 2.3. Experimental and Simulated Raman and ROA Spectra of ph-β-Glu and Gastrodin in Water

**Figure 8:**
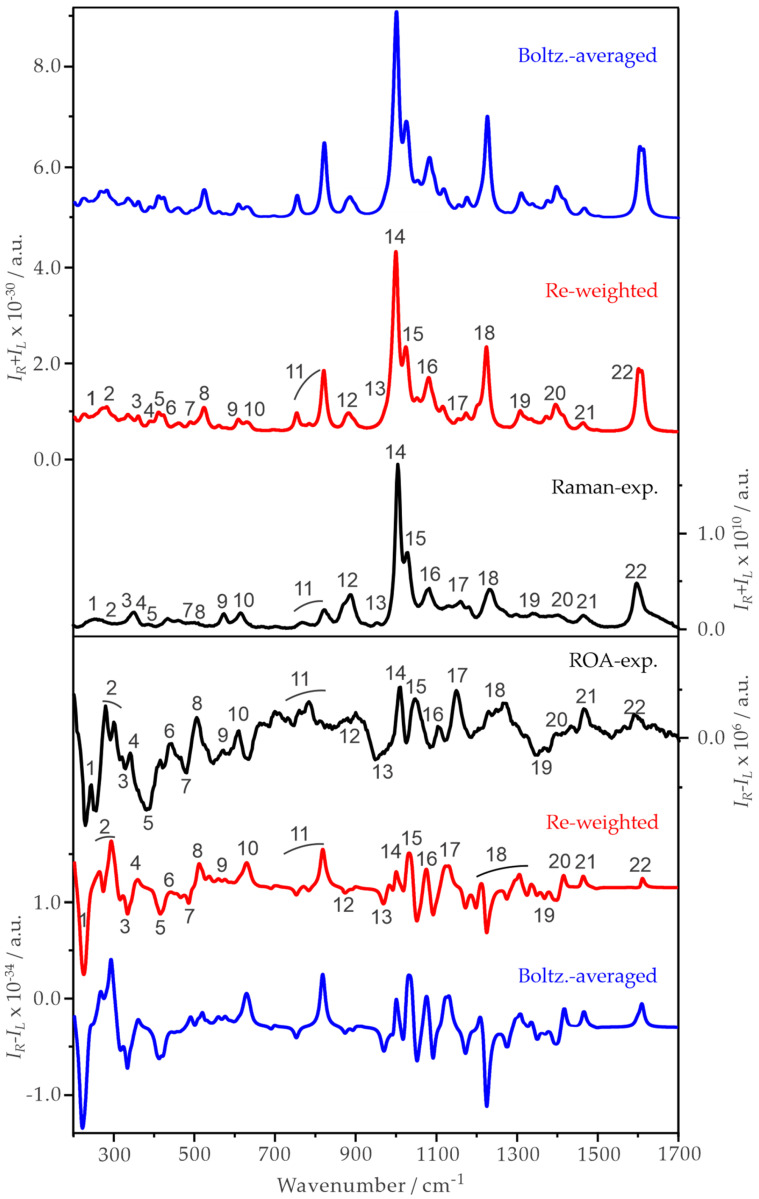
Comparison of the experimental Raman and ROA spectra of ph-β-glu in water with the corresponding Boltzmann averaged and re-weighted Raman and ROA spectra, simulated at the B3LYP-D3BJ/def2-TZVPD level with the PCM (water). The predicted Boltzmann percentage factors are listed in Figure 2 and the adjusted Boltzmann factors are described in the main text. The main Raman and ROA features are numbered to facilitate easy comparison.

The individual conformer Raman and ROA spectra of the 14 most stable ph-β-glu conformers at the B3LYP-D3BJ/def2-TZVPD/PCM (water) level of theory are depicted in Figure S5, Supplementary Materials. Both Raman and ROA spectra exhibit some variations from one conformer to the next, although the changes are not as severe as what were observed in the case of VCD features of ph-β-glu discussed in the previous section. In Figure 8, the Boltzmann weighted Raman and ROA spectra are given, with the Boltzmann percentage factors of all the individual conformers in water listed in Figure 3. Compared to the conformational distribution of ph-β-glu in DMSO, its distribution in water is more spread out with many more conformers which were predicted to contribute nearly equally, making detailed band assignment more challenging.

In general, the Boltzmann averaged and the experimental Raman spectra of ph-β-glu in water exhibit good agreement. While the experimental and simulated ROA spectra agree overall, a closer examination of the individual ROA spectra suggests that some improvement can be made by emphasizing the contribution of several conformers than what were indicated by their theoretical Boltzmann factors. The empirical weighting factors were chosen based on the same general principle discussed in the previous section and their values are as follows: 20% for G-g+/cc/T, 30% for G+g-/cc/T, 5% for Tg+/cc/T, 10% for G-g-/cc/T, 5% for the next four most stable conformers: G-g+/c/T, G+t/cc/T, G-t/cc/T, and G+g+/cc/T, 3% for G+g-/c/T, 1% for Tt/c/T and Tg-/c/T, and finally 10% for G+g-/cc/G-. Generally, these percentage values in water differ somewhat from those in DMSO, a point which we will further address in Section 2.4. The percentage changes are relatively small compared to the calculated Boltzmann percentages, except that the weight of G+g-/cc/G- was increased noticeably because it reproduced the ROA feature around 500 cm^−1^ better than others. The resulting re-weighted Raman and ROA spectra are also presented in Figure 8. While the Boltzmann versus re-weighted Raman spectra appear essentially unaltered, the re-weighted ROA spectrum exhibits some noticeable improvements in the whole frequency range, highlighting the specific conformational sensitivity of ROA. For example, the ROA pattern in the lower wavenumber region for the bands 1 to 10 is much better reproduced, as is the relative intensity of most bands in the whole frequency region.

**Figure 9:**
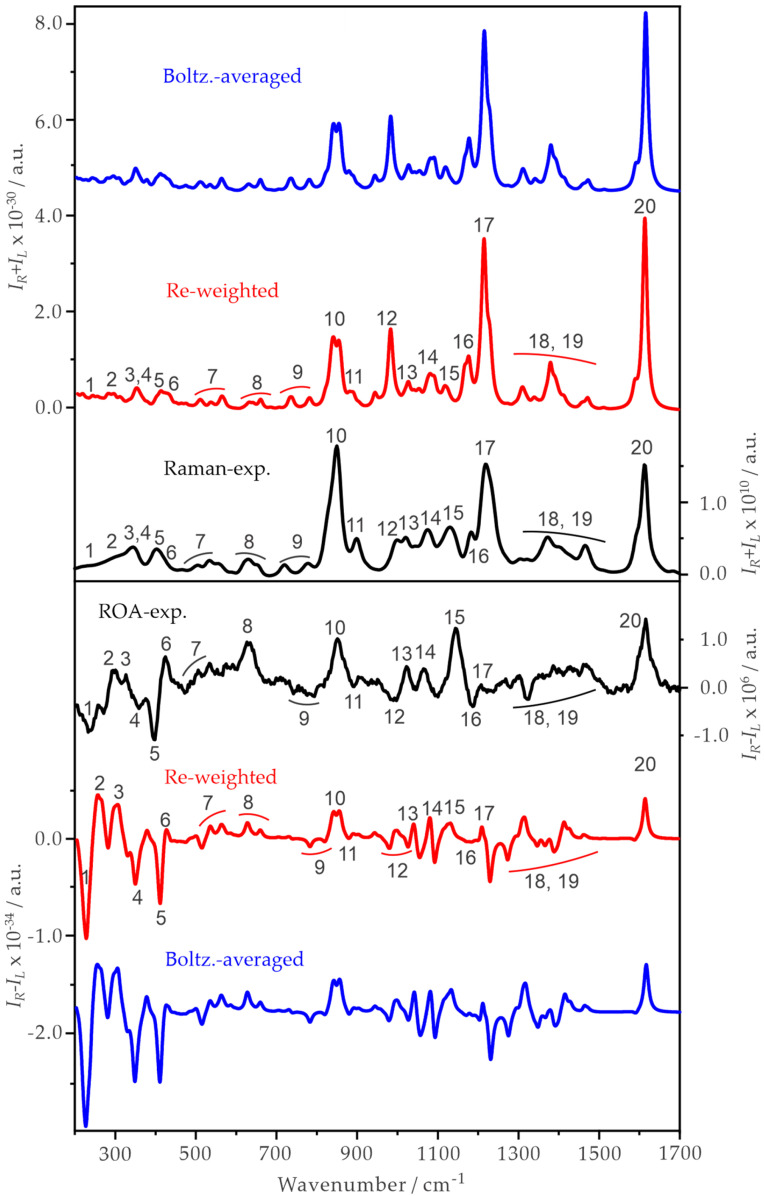
Comparison of the experimental Raman and ROA spectra of gastrodin in water with the corresponding Boltzmann averaged and re-weighted Raman and ROA spectra, simulated at the B3LYP-D3BJ/def2-TZVPD level with the PCM (water). The predicted Boltzmann percentage factors are listed in Figure 3 and the adjusted Boltzmann factors are described in the main text. The main ROA and the corresponding Raman features are numbered for easy comparison. See the main text for discussions.

For gastrodin, the individual conformer Raman and ROA spectra of the 19 most stable conformers at the B3LYP-D3BJ/def2-TZVPD/PCM (water) level of theory are presented in Figure S6, Supplementary Materials. The Boltzmann averaged Raman and ROA spectra of gastrodin, using the Boltzmann percentage factors listed in Figure 3, are presented in Figure 9, as well as the re-weighted Raman and ROA spectra. Since gastrodin has a large number of low-energy conformers, small adjustments were made to the percentage abundances of a number of the lowest energy conformers, whereas the rest of the conformers were held at the predicted values. The changes made are: 10% for G-g+/cc/T/G+g-, 10% for G+g-/cc/T/G-g-, 8% for G+g-/cc/T/G+g+, 10% G+g-/cc/T/G-g+, 12% for G-g+/cc/T/G-g-, 4% for G+g-/cc/T/G+g-, 12% for G-g+/cc/T/G+g+, 10% for G-g+/cc/T/G-g+, and 7% for G+g+/cc/T/G+g-.

To facilitate the detailed comparison, the main ROA and the corresponding Raman features of gastrodin in water were numbered from 1 to 20. Because of the overlapping bands, a Raman band peak does not necessarily correspond to a ROA band peak, making it occasionally difficult to correlate the experimental Raman and ROA bands. For the simulated ROA and Raman bands, on the other hand, it is easy to line up the corresponding ROA and Raman features. We utilized this corresponding relationship extensively in assigning the experimental ROA and the related Raman bands.

Overall, the simulated and experimental Raman and ROA spectra agree well with each other, while the re-weighted spectra offer somewhat better agreement with the experiment. For example, the relative intensities of the ROA bands labelled as four to six were better captured in the re-weighted spectra. For the Raman spectra, the simulated partially resolved double bands labelled as “10” may be used to explain the appearance of lower band intensity compared to the corresponding experimental intensity. Based on the good agreement achieved for both Raman and ROA, we can conclude that the important gastrodin conformers in water with percentage abundances over 6% are: G-g+/cc/T/G+g-, G+g-/cc/T/G-g-, G+g-/cc/T/G+g+, G+g-/cc/T/G-g+, G-g+/cc/T/G-g-, G-g+/cc/T/G+g+, G-g+/cc/T/G-g+, and G+g+/cc/T/G+g-, strongly favoring the gauche conformations of the hydroxymethyl group at the pyranose ring.

### 2.4. Some General Comments about the Conformational Distribution of ph-β-Glu and Gastrodin in DMSO and in Water

Anomeric effects associated with carbohydrate chemistry are of continuous research interest and have recently been reviewed [42,43]. When α or β-D-glucopyranosides are dissolved in water, an equilibrium is established with about 36% α and 64% β-D-glucopyranosides at room temperature, and this conformational equilibrium can be further influenced by the solvent(s) used [42,44]. In the two β-anomers studied here, their glycosides have “protected” anomeric centers due to the substitution at the O1 position and they do not undergo mutarotation in DMSO or in water. This is why we do not need to be concerned about such an equilibrium in the current study. We would expect to observe the equilibrium between the β- and α-anomers of these two compounds if, for example, an acidic condition was provided [45].

With the combined experimental and theoretical results obtained for these two carbohydrates in DMSO and in water, we can now compare the conformational distributions obtained in these two different solvents and in the gas phase when available. For ph-β-glu in DMSO, G-g+/cc/T (60%) is by far the most important conformer, followed by G+g-/cc/T, Tg+/cc/T, and G-g-/cc/T (about 10% each). In the previous IR-UV hole-burning spectroscopic study of ph-β-glu, only three conformers were observed experimentally, namely G+g-/cc/T, G-g+/cc/T, and Tg+/cc/T, with 68%, 25%, and 7% abundances, respectively [11]. No other less-stable conformers were detected experimentally in the gas phase. Although the same three conformers are also among the most abundant conformers of ph-β-glu identified in DMSO and in the gas phase, the most abundant conformer in DMSO is G-g+/cc/T (60%) in contrast to G+g-/cc/T (68%) identified in the gas phase. This observation highlights the noticeable solvent influence on the relative stability of the ph-β-glu conformers. If one groups the abundances based on the hydroxymethyl (at the pyranose ring) conformations G+:G-:T, the values in DMSO are 15%:75%:10%, respectively, emphasizing the presence of T conformation, as was the case in the previous gas phase study [11].

For ph-β-glu in water, the four main conformers identified in DMSO are still important, but the conformational distribution becomes more widely spread with many more conformers contribute above 5%. The four most abundant ones are G-g+/cc/T (20%), G+g-/cc/T (30%), G-g-/cc/T (10%), and G+g-/cc/G- (10%). In the current experiments, we have the percentage abundances of the hydroxymethyl conformations G+:G-:T in water to be 53%:40%:7%, which is very different from the distribution in DMSO.

For gastrodin, there have been no detailed conformational studies reported in the gas phase or in solution. We therefore focus on the comparison of its conformational distribution in DMSO and in water, as well as the comparison to ph-β-glu. The main gastrodin conformers in water and in DMSO are in general similar, with some modest variation in their individual abundances. If we group together the gastrodin conformers based on whether their ph-β-glu part takes on the G+g-/cc/T, G-g+/cc/T, G+g+/cc/T, Tg+/cc/T, or Tt/cc/T conformations, the corresponding abundances are 47%, 22%, 9%, 8%, or 14% in DMSO, respectively, versus 61%, 21%, 9%, 8%, and 0% (~0.3%) in water. These numbers can be further grouped into the abundances based on the hydroxymethyl (at the pyranose ring) conformations G+:G-:T, which are 56%, 22%, and 22% for gastrodin in DMSO and 70%, 21%, and 8% for gastrodin in water. Again, the preference for the T conformation of the hydroxymethyl group at the pyranose ring was enhanced in DMSO compared to in water, while a similar trend was also observed with ph-β-glu in the discussion above.

The re-weighted percentage abundances to better reproduce the experimental IR, VCD, Raman, and ROA spectra differ somewhat from the theoretical Boltzmann factors while generally following the predicted stability trend. Such deviation is likely caused by some explicit solvent effects which have not been taken into account in the current modeling. However, in a recent IR and VCD study of methyl-β-D-glucose in water by some of the authors [46], we attempted solvation with multiple water molecules with the help of the newly developed Quantum Cluster Growth (QCG) program [47]. The choice to stay with the implicit solvation model was made because of the complexity of the two molecular targets and the challenge to sample all important solvation positions of solvent molecules like water. Recently, ab initio molecular dynamics (AIMD) simulations have become possible for VCD [48,49] and ROA [50,51], although their applications are still limited because of the high computing cost. Nevertheless, the AIMD VCD and ROA simulations of lactic acid and N-acetyl-L-cysteine, both of which are flexible chiral molecules in water, were recently reported [48,50]. Future work by using AIMD for VCD and ROA of ph-β-glu and gastrodin would be of significant interest for further exploration of the solvation effects.

## 3. Materials and Methods

### 3.1. Experimental

The gastrodin and ph-β-glu (both purity ≥ 98.0%) were purchased from Sigma-Aldrich and used without further purification. Deuterated dimethyl sulfoxide and methanol were also purchased from Sigma Aldrich, St. Louis, MO, USA, and used as they were. All IR and VCD spectra were collected using a FTIR spectrometer (Bruker Vertex 70, Billerica, MA, USA) coupled to a VCD model (PMA 50). The photoelastic modulator (PEM) was set at 1400 cm^−1^ for all measurements. The liquid nitrogen cooled mercury cadmium telluride (MCT) detector was used, and the resolution was set at 4 cm^−1^. The DMSO solutions of the gastrodin and ph-β-glu were prepared with a concentration of 0.35 M and 0.80 M, respectively. A demountable BaF_2_ cell with a 0.05 mm Teflon space was used for all measurements, and the collection time was two hours for each. The final IR and VCD spectra were baseline corrected by the subtraction of the solvent spectrum measured under the same conditions.

The aqueous solutions of gastrodin and ph-β-glu were prepared with a concentration of 0.70 M. The Raman and ROA spectra of gastrodin and ph-β-glu in water were measured using a ChiralRaman-2X^TM^ spectrometer (BioTools, Jupiter, FL, USA). The spectra were collected in the 200~2000 cm^−1^ region using a 532 nm laser excitation source and the sample was irradiated using a laser power of 200 mW (at the source) for about 48 h.

### 3.2. Theoretical

To systematically explore the conformational landscapes of ph-β-glu and gastrodin, we utilized the CREST code [24] by Grimme and co-workers with the inclusion of the generalized Born (GB)-based GBSA implicit solvation model [32,33], using DMSO and water as the solvents. Built upon the previous semiempirical tight-binding (TB) quantum chemistry method, called GFN-xTB [52], the new CREST code is capable of the fast and reliable exploration and screening of the conformational space of mid- to large-sized molecules with up to about a thousand atoms. To reduce computational costs, we followed the multitiered approach developed before [34]: (i) searched for all the CREST candidates; (ii) performed a relaxed geometry optimization at the revPBE-D3/def2-SVP level [53], with the empirical D3 dispersion correction for the CREST candidates, followed by a single-point energy evaluation at the B3LYP-D3/def2-TZVP level of the optimized structures. This step was done using Molpro [54], and the purpose was to narrow the energy window to 15 kJ mol^−1^ without losing any important conformers. The relative conformational energies obtained at this step were shown to correlate well with the results obtained with the final DFT calculations at a higher level of theory [34]; (iii) carried out the final geometry optimization and harmonic frequency calculations using the Gaussian 16 package [55]. In the current study, the simulations of IR, VCD, Raman, and ROA spectra were done at the B3LYP-D3BJ/def2-TZVP and def2-TZVPD levels, although minimal differences were observed between them. All results presented are at the latter level. The implicit solvent was included using the integral equation formalism (IEF) version of the PCM [56] to account for the bulk DMSO and water solvent environment.

A Lorentzian band shape with a half-width at half-height (HWHH) of 4 cm^−1^ was applied to the simulations of IR, VCD, Raman, and ROA spectra. We also applied a linear correlation method proposed in Ref. [57] to scale the simulated frequencies in the current study. This procedure was applied recently in a study of transition metal complexes [58] and was shown to facilitate better comparison with the experimental IR and VCD spectra.

## 4. Conclusions

The conformational distributions of ph-β-glu and gastrodin in DMSO and in water were investigated using a combined experimental and theoretical approach with IR, VCD, Raman, and ROA spectroscopies. A large number of conformational candidates were generated with the systematic CREST searches, which ensured a proper exploration of the complicated conformational landscapes of these two carbohydrates. In general, good agreements between the experimental and simulation IR, VCD, Raman, and ROA spectra at the B3LYP-D3BJ/def2-TZVPD level were achieved. It is noted that the VCD and ROA spectral features vary greatly among different conformers, whereas the parent IR and Raman features exhibit much less variation. Consequently, the dominant conformations of ph-β-glu and gastrodin in DMSO and in water were extracted experimentally based mainly on the comparison of the simulated and experimental VCD and ROA spectra, with the guidance of theoretical conformational stability trends. Since ph-β-glu and gastrodin share the same core ph-β-glu part, it is interesting to compare the changes induced by the extra substitution of the hydroxymethyl group at the para-position of the phenyl ring. It is particularly gratifying to note that the experimental variation observed in the IR and VCD spectra of these two compounds are very well reproduced theoretically, confirming the good quality of the current theoretical modelling. Furthermore, the current study shows that the conformational abundances based on the hydroxymethyl (at the pyranose ring) conformations, i.e., G+:G-:T, are 15%:75%:10% in DMSO, in contrast to 68%:25%:7% in the gas phase [11] and 53%:40%:7% in water, respectively, emphasizing the importance of the solvent effects. For gastrodin, the aforementioned percentage abundances are 56%, 22%, and 22% in DMSO and 70%, 21%, and 8% in water. The abundance the T conformation was shown to be enhanced in DMSO compared to in water, similar to the situation with ph-β-glu. The current work showcases the power of using multiple chiroptical tools, i.e., VCD and ROA, aided by theoretical calculations, in exploring conformational distributions of carbohydrate derivatives directly in solution.

## Figures and Tables

**Table 1:** The parameters of four main conformers of ph β-glu at the B3LYP-D3BJ/def2-TZVPD level in DMSO.

Structural Parameters	G-g+/cc/T	G+g-/cc/T	Tg+/cc/T	G-g-/cc/T
r(H_2_-O_1_)/Å	2.583	2.590	2.583	2.582
r(H_3_-O_2_)/Å	2.528	2.540	2.551	2.542
r(H_4_-O_3_)/Å	2.426	2.420	2.471	2.419
r(H_6_-O_4_/O_5_)/Å	2.423	2.407	1.994	3.060
θ(C_2_-C_1_-O_1_-C_7_)/°	160.1	161.2	160.0	160.1
θ(C_3_-C_2_-O_2_-H_2_)/°	−172.4	−172.3	−172.2	−172.5
θ(C_4_-C_3_-O_3_-H_3_)/°	176.8	175.8	176.3	176.6
θ(C_5_-C_4_-O_4_-H_4_)/°	173.3	171.9	179.3	172.5
θ(O_5_-C_5_-C_6_-O_6_)/°	−60.2	61.6	169.7	−66.5
θ(C_5_-C_6_-O_6_-H_6_)/°	57.2	−56.6	48.2	−82.1
θ(C_1_-O_1_-C_7_-C_12_)/°	14.8	12.6	14.8	14.4

**Table 2:** The parameters of four main conformers of gastrodin at the B3LYP-D3BJ/def2-TZVPD level in DMSO.

Structural Parameters	G-g+/cc/T/G+g-	G+g-/cc/T/G-g-	G+g-/cc/T/G+g+	G+g-/cc/T/G-g+
r(H_2_-O_1_)/Å	2.596	2.595	2.596	2.593
r(H_3_-O_2_)/Å	2.531	2.529	2.532	2.531
r(H_4_-O_3_)/Å	2.409	2.412	2.409	2.411
r(H_6_-O_5_)/Å	2.405	2.400	2.403	2.401
r(H_12_-O_5_)/Å	2.553	2.558	2.556	2.550
θ(C_2_-C_1_-O_1_-C_7_)/°	161.6	161.5	161.6	161.1
θ(C_3_-C_2_-O_2_-H_2_)/°	−171.6	−171.6	−171.6	−171.7
θ(C_4_-C_3_-O_3_-H_3_)/°	176.2	176.4	176.2	176.2
θ(C_5_-C_4_-O_4_-H_4_)/°	171.2	171.4	171.2	171.4
θ(O_5_-C_5_-C_6_-O_6_)/°	−60.2	61.4	61.4	61.4
θ(C_5_-C_6_-O_6_-H_6_)/°	57.4	−57.1	−57.3	−57.2
θ(C_1_-O_1_-C_7_-C_12_)/°	12.5	12.7	12.5	13.3
θ(C_9_-C_10_-C_13_-O_7_)/°	101.2	−73.4	74.6	−100.9
θ(C_10_-C_13_-O_7_-H_7_)/°	−56.4	−56.4	56.4	56.7

## Data Availability

Data are contained within the article.

## Supplementary Materials

The following supporting information can be downloaded at: https://www.mdpi.com/article/10.3390/molecules28104013/s1. Figures S1 and S2: The C1-O1-C7-C12 dihedral angle values of the 14 most stable ph-β-glu conformers and of the 19 most stable gastrodin conformers; Figure S3: Geometries of the five gastrodin conformers; Figure S4: Simulated IR and VCD spectra of the most stable gastrodin conformers; Figure S5: Simulated Raman and ROA spectra of the most stable ph-β-glu conformers; Figure S6: Simulated Raman and ROA spectra of the most stable gastrodin conformers. Tables S1 and S2: The parameters of four main conformers of ph-β-glu and of gastrodin at the B3LYP-D3BJ/def2-TZVPD level in water.

## Sample Availability

They are commercially available from Sigma Aldrich.
