# Supplementary material for: Conformational Distributions of Phenyl β-D-Glucopyranoside and Gastrodin in Solution by Vibrational Optical Activity and Theoretical Calculations
Source: Molecules. 2023 May 10;28(10):4013. doi: 10.3390/molecules28104013 (PMC10222027; doi:10.3390/molecules28104013)
Supplement: Supplementary file 1 [file molecules-28-04013-s001.zip › molecules-2382831-supplementary.pdf]

molecules

Supplementary Material

Conformational Distributions of Phenyl β-D-Glucopyranoside and Gastrodin in Solution by Vibrational Optical Activity and Theoretical Calculations

Mutasem Alshalalfeh [1], Ningjie Sun [1,2], Amanda Hanashiro Moraes [1], Alexandra Paola Aponte Utani [1], and Yunjie Xu [1,*]

1 Department of Chemistry, University of Alberta, Edmonton, AB T6G 2G2, Canada; alshalal@ualberta.ca (M.A.); hanashir@ualberta.ca (A.H.M.); aponteut@ualberta.ca (A.P.A.U.)

2 State Key Laboratory of Advanced Metallurgy, University of Science and Technology Beijing, 100083 Bei-jing, China; b20180539@xs.ustb.edu.cn (N.S.)

* Correspondence: yunjie.xu@ualberta.ca; Tel.: +1-780-492-1244

Contents

Figure S1. The C1-O1-C7-C12 dihedral angle values of the 14 most stable ph-β-glu conformers S2

Figure S2. The C1-O1-C7-C12 dihedral angle values of the 19 most stable gastrodin conformers S3

Figure S3. Geometries of the five less stable conformers of gastrodin S4

Figure S4. The simulated individual IR and VCD spectra of the 19 most stable gastrodin conformers S5

Figure S5. The simulated individual Raman and ROA spectra of the 14 most stable ph-β-glu conformers S6

Figure S6. Simulated individual Raman and ROA spectra of the 19 most stable gastrodin conformers S7

Table S1. The parameters of four main conformers of ph β-gluin in water S8

Table S2. The parameters of four main conformers of gastrodin in water. S8

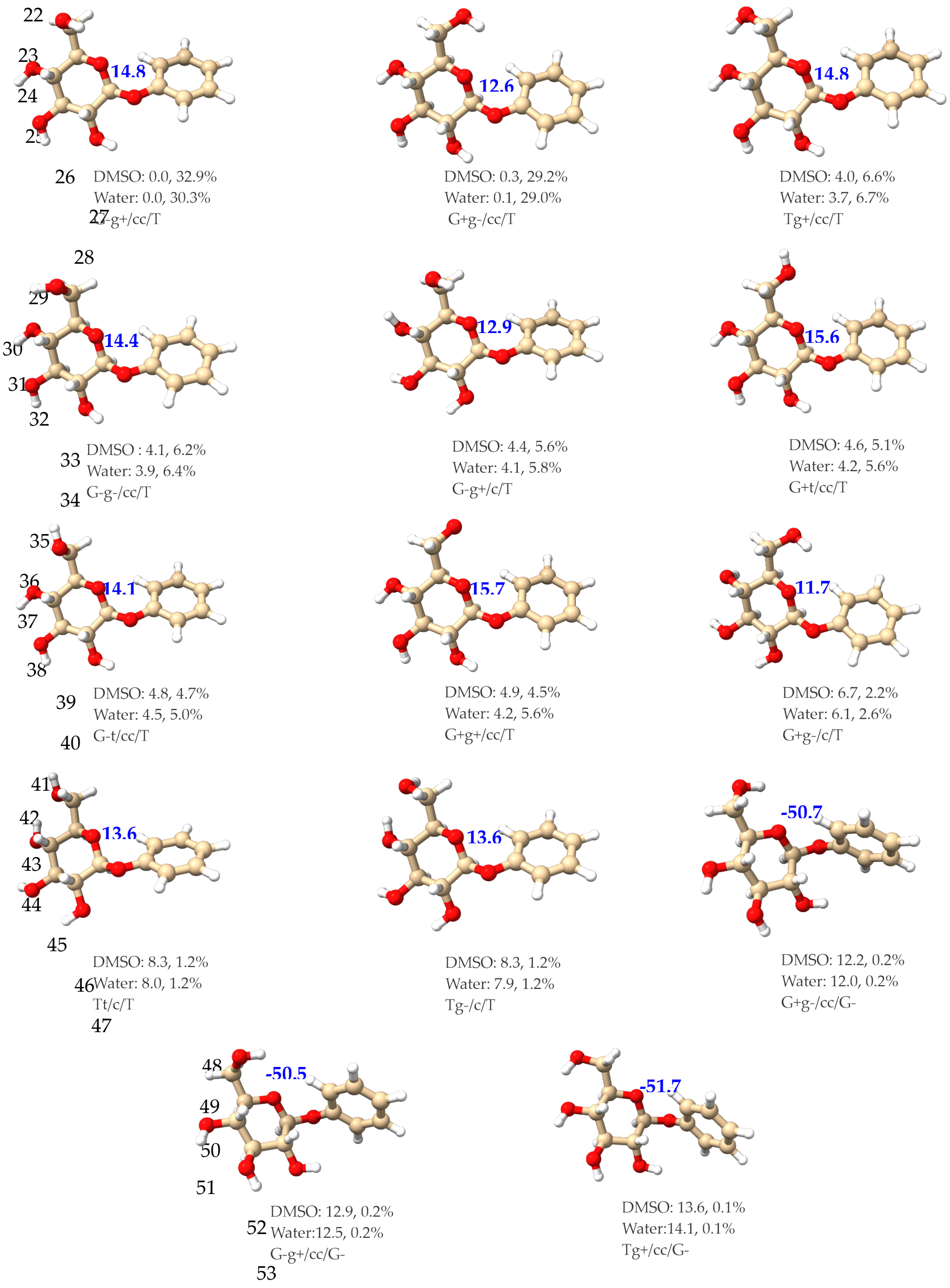

Figure S1. The C1-O1-C7-C12 dihedral angle values which correspond to the tilting of the phenyl plane are given in blue (in unit of degree) for each of the 14 most stable conformers of ph-β-glu at the B3LYP-D3BJ/def2-TZVPD level with the PCM of DMSO.

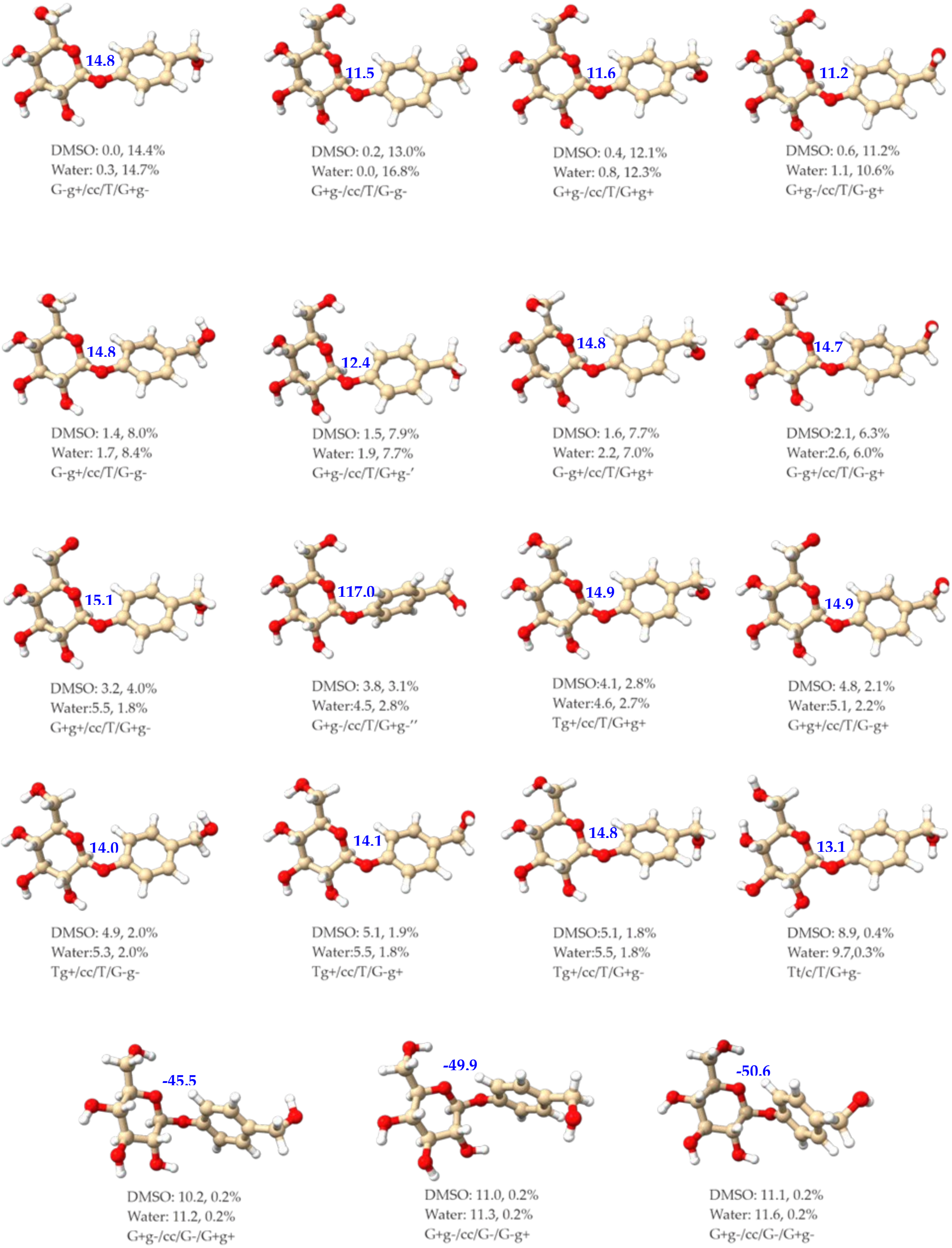

Figure S2. The C1-O1-C7-C12 dihedral angle values which correspond to the tilting of the phenyl plane are given in blue (in unit of degree) for each of the 19 most stable conformers of gastrodin at the B3LYP-D3BJ/def2-TZVPD level with the PCM of DMSO.

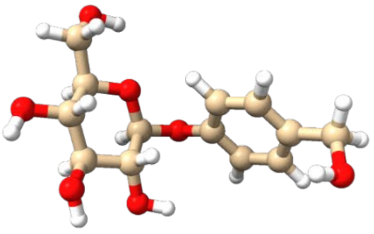

DMSO: 11.4, 0.1%
Water: 11.8, 0.1%
G+g-/cc/G-/G-g-

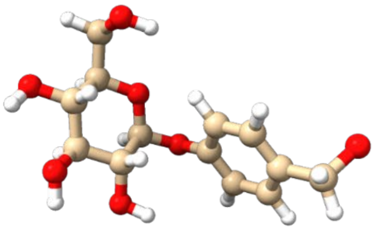

DMSO: 11.8, 0.1%
Water: 11.8, 0.1%
G-g+/cc/G-/G+g-

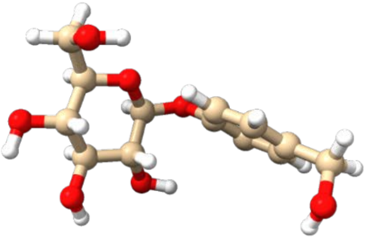

DMSO: 13.3, 0.1%
Water: 13.6, 0.1%
G-g+/cc/G-/G-g+

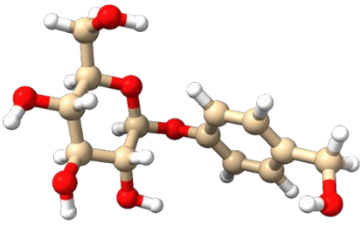

DMSO: 13.7, 0.1%
Water: 14.2, 0.1%
G-g+/cc/G-/G-g-

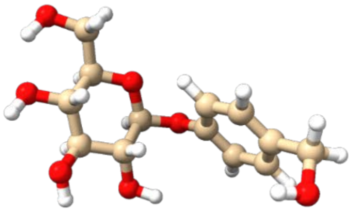

DMSO: 13.9, 0.1%
Water: 14.2, 0.1%
Tg+/cc/G-/G-g-

Figure S3. Geometries of the five less stable conformers of gastrodin with the Boltzmann population factors ≤ 0.1% at the B3LYP-D3BJ/def2-TZVPD level with the PCM of DMSO or Water. The relative free energies in kJ mol^{-1} and the percentage Boltzmann factors at 298 K are also given for both solvents.

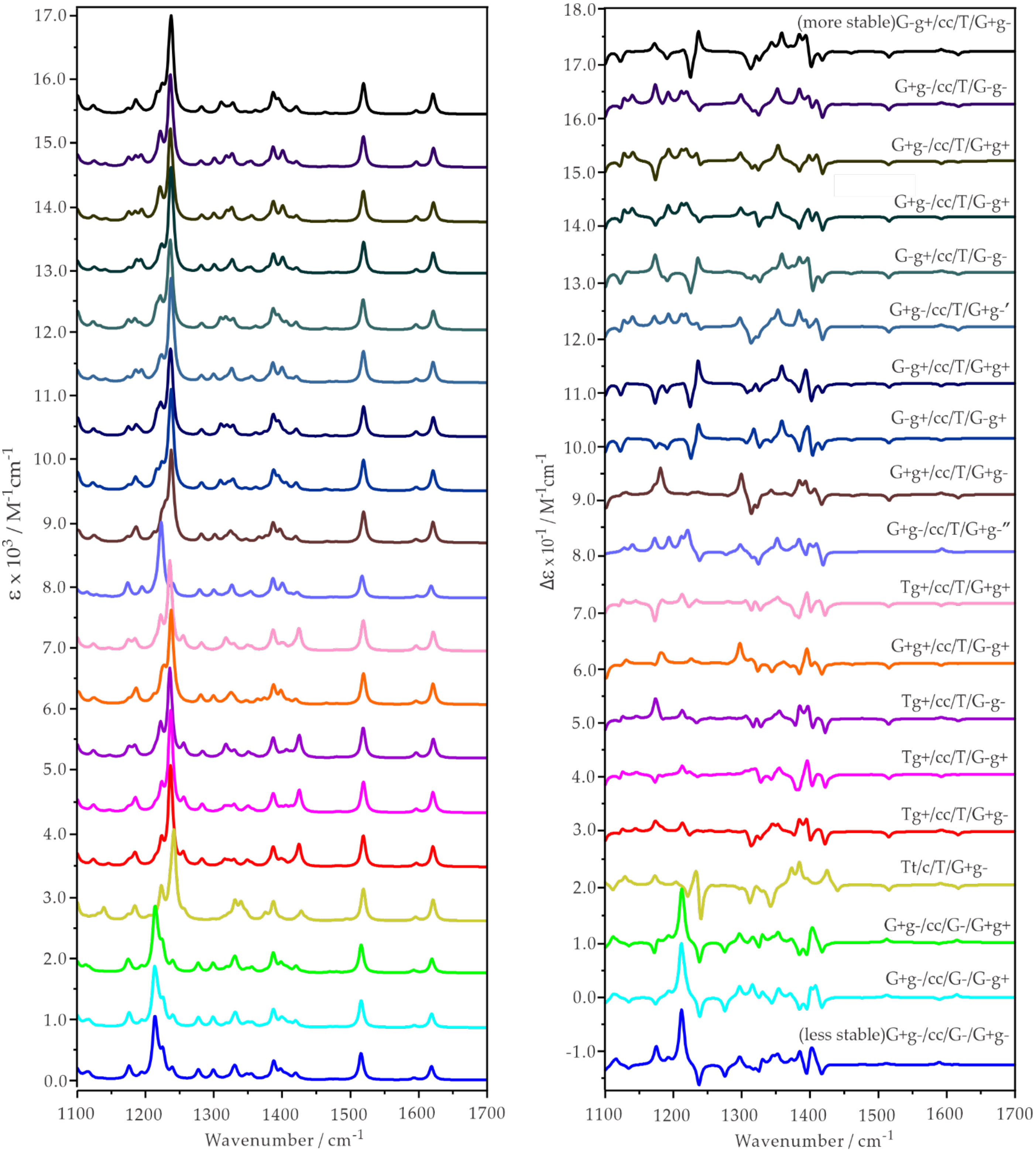

Figure S4. The simulated individual IR and VCD spectra of the 19 most stable gastrodin conformers with the Boltzmaan population factors greater than 0.1% at the B3LYP-D3BJ/def2-TZVPD level with the PCM of DMSO.

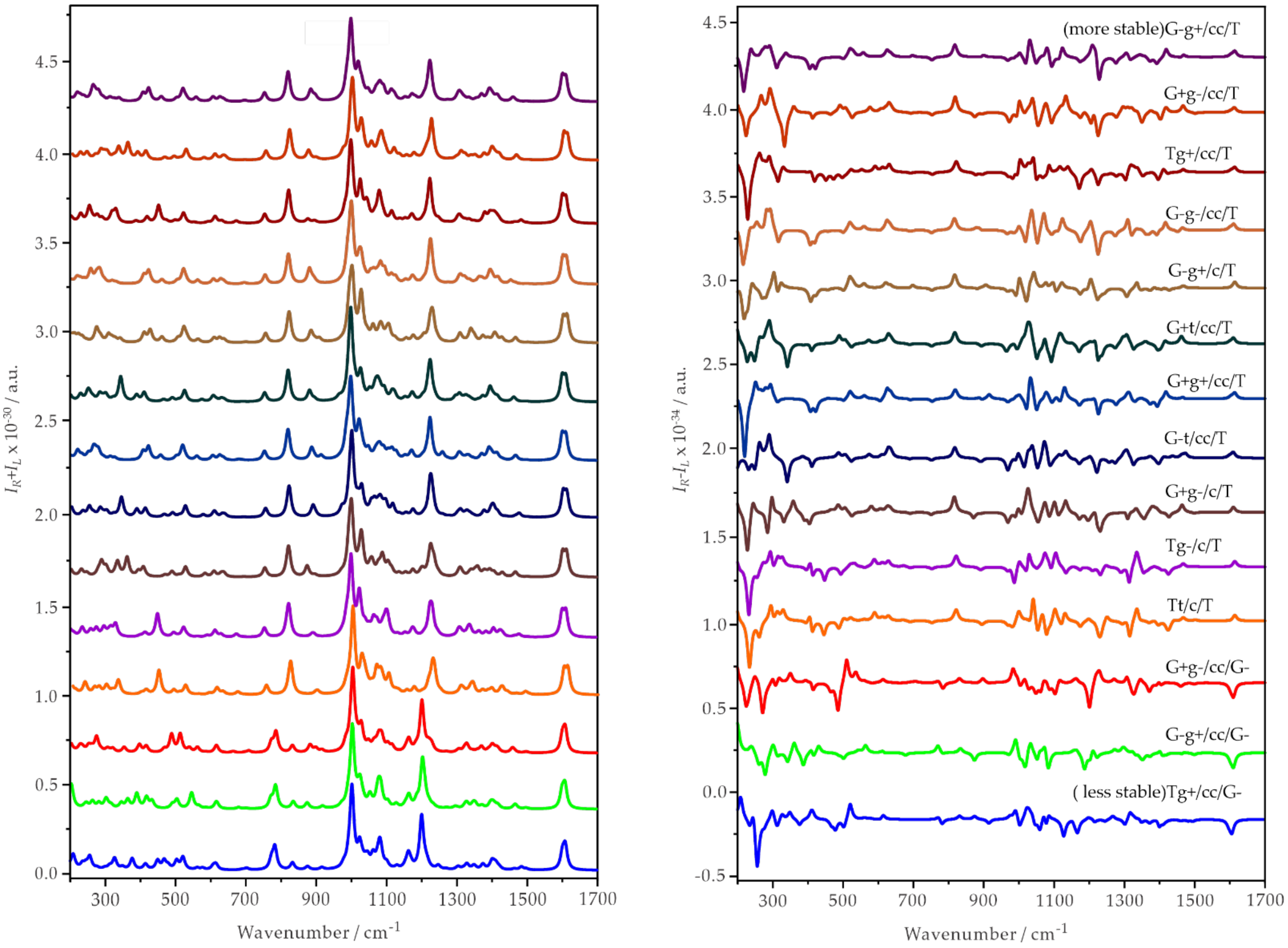

Figure S5. The simulated individual Raman and ROA spectra of the 14 most stable ph-β-glu conformers at the B3LYP-D3BJ/def2-TZVPD level with the PCM of water.

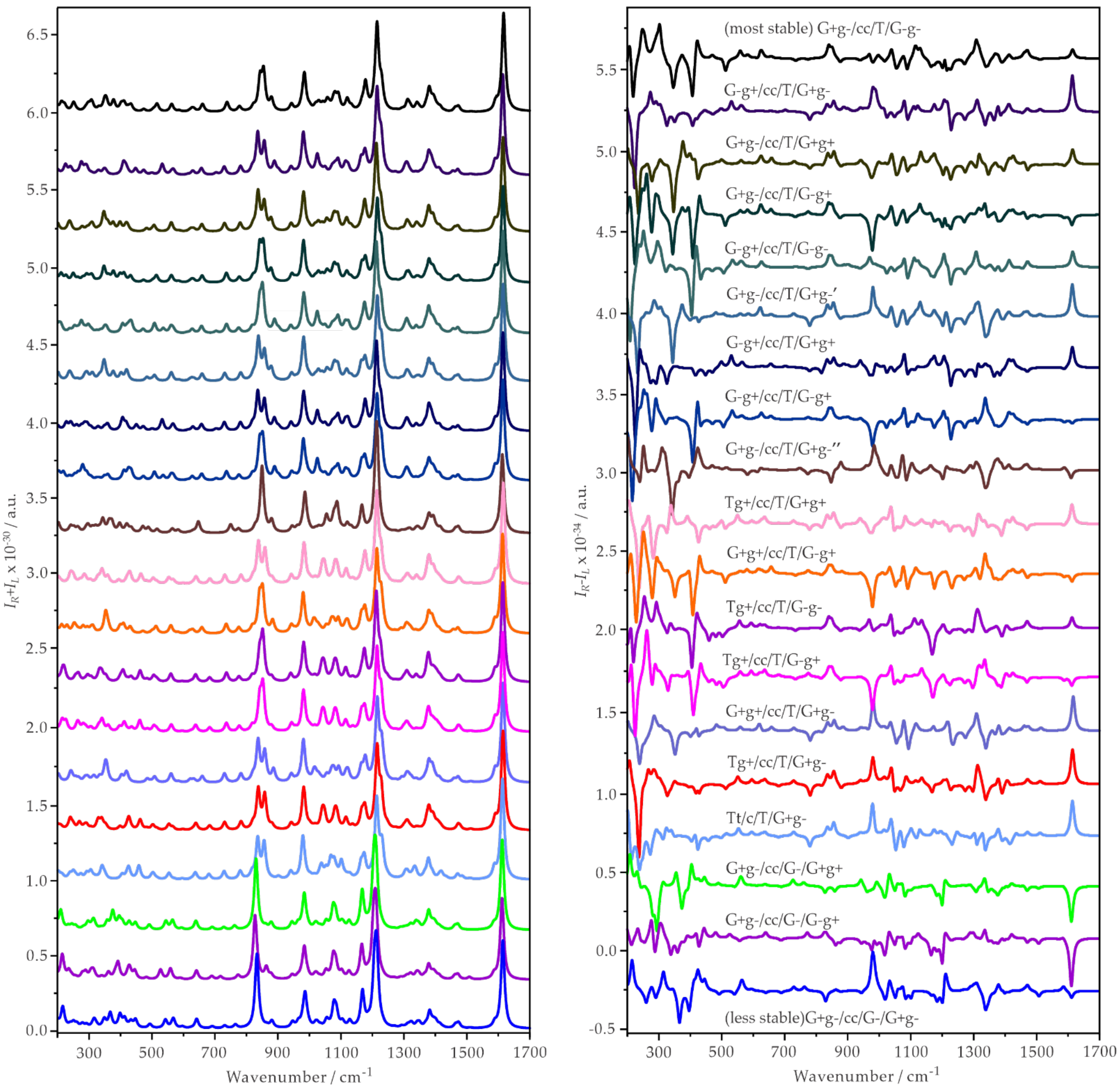

Figure S6. The simulated individual Raman and ROA spectra of the 19 most stable gastrodin conformers with the Boltzmaan population factors greater than 0.1% at the B3LYP-D3BJ/def2-TZVPD level with the PCM of water.

Table S1. The parameters of four main conformers of ph β-glu at the B3LYP-D3BJ/def2-TZVPD level in water.

Structural parameters	**G-g+/cc/T**	**G+g-/cc/T**	**Tg+/cc/T**	**G-g-/cc/T**
$r(H_2\text{-}O_1)$/Å	2.584	2.591	2.584	2.583
$r(H_3\text{-}O_2)$ /Å	2.532	2.542	2.554	2.544
$r(H_4\text{-}O_3)$ /Å	2.424	2.420	2.474	2.420
$r(H_6\text{-}O_4/O_5)$ /Å	2.426	2.408	1.993	3.067
$\theta(C_2\text{-}C_1\text{-}O_1\text{-}C_7)$/°	160.0	161.2	160.0	160.1
$\theta(C_3\text{-}C_2\text{-}O_2\text{-}H_2)$ /°	-172.4	-172.1	-172.2	-172.4
$\theta(C_4\text{-}C_3\text{-}O_3\text{-}H_3)$ /°	176.5	175.5	176.1	176.3
$\theta(C_5\text{-}C_4\text{-}O_4\text{-}H_4)$ /°	173.0	171.9	179.6	172.6
$\theta(O_5\text{-}C_5\text{-}C_6\text{-}O_6)$ /°	-60.3	61.7	169.8	-66.3
$\theta(C_5\text{-}C_6\text{-}O_6\text{-}H_6)$ /°	57.3	-56.5	48.2	-82.2
$\theta(C_1\text{-}O_1\text{-}C_7\text{-}C_{12})$ /°	14.9	12.7	14.9	14.5

Table S2. The parameters of four main conformers of gastrodin at the B3LYP-D3BJ/def2-TZVPD level in water.

Structural parameters	**G-g+/cc/T/G+g-**	**G+g-/cc/T/G-g-**	**G+g-/cc/T/G+g+**	**G+g-/cc/T/G-g+**
$r(H_2\text{-}O_1)$/Å	2.587	2.595	2.594	2.596
$r(H_3\text{-}O_2)$ /Å	2.531	2.542	2.541	2.541
$r(H_4\text{-}O_3)$ /Å	2.422	2.417	2.418	2.416
$r(H_6\text{-}O_5)$ /Å	2.425	2.416	2.413	2.411
$r(H_{12}\text{-}O_5)$ /Å	2.546	2.577	2.576	2.577
$\theta(C_2\text{-}C_1\text{-}O_1\text{-}C_7)$/°	160.3	161.6	161.5	161.6
$\theta(C_3\text{-}C_2\text{-}O_2\text{-}H_2)$ /°	-172.2	-171.9	-171.9	-171.8
$\theta(C_4\text{-}C_3\text{-}O_3\text{-}H_3)$ /°	176.5	175.5	175.6	175.5
$\theta(C_5\text{-}C_4\text{-}O_4\text{-}H_4)$ /°	172.9	171.7	171.9	171.7
$\theta(O_5\text{-}C_5\text{-}C_6\text{-}O_6)$ /°	-60.3	61.8	61.9	61.8
$\theta(C_5\text{-}C_6\text{-}O_6\text{-}H_6)$ /°	57.4	-57.0	-56.8	-57.0
$\theta(C_1\text{-}O_1\text{-}C_7\text{-}C_{12})$ /°	14.5	11.5	11.5	11.3
$\theta(C_9\text{-}C_{10}\text{-}C_{13}\text{-}O_7)$ /°	100.2	-74.1	74.9	-100.6
$\theta(C_{10}\text{-}C_{13}\text{-}O_7\text{-}H_7)$ /°	-56.8	-56.6	56.6	56.9
